# Selective attentional bias to food-related stimuli in healthy individuals with characteristics towards orthorexia nervosa

**DOI:** 10.1007/s40519-019-00755-z

**Published:** 2019-07-27

**Authors:** Ian P. Albery, Monika Michalska, Antony C. Moss, Marcantonio Spada

**Affiliations:** Centre for Addictive Behaviours Research, School of Applied Sciences, London South Bank University, London, UK

**Keywords:** Orthorexia nervosa, Attentional bias, ORTO-15, Stroop-task

## Abstract

**Purpose:**

It has been argued that orthorexia nervosa (ON) is a unique type of disordered eating of food considered by the individual to be healthy. Given that in other eating disorder populations attentional preference for food-related cues influences eating behaviours, is it also likely that these biases may be a characteristic of ON tendency.

**Methods:**

Eighty healthy individuals completed the ORTO-15 questionnaire (ON tendency), a modified Stroop task containing words related to healthy and unhealthy foods and perceived hunger levels pre- and post-testing. The ORTO-15 was used to identify participants within this sample who demonstrated more or less of the characteristics of ON.

**Results:**

Results suggest that the presence of attentional bias to healthy, but not for unhealthy food-related stimuli independently predict increased ON tendency. Increased attentional bias towards healthy food-related stimuli is associated with increased scores on the ORTO-15.

**Conclusion:**

Attentional bias, as a deficit in information processing, towards healthy food-related stimuli accounts for variability in ON characteristics.

**Level of evidence:**

Level I, experimental study.

## Introduction

Orthorexia nervosa (ON) is characterized by obsessive and selective eating behaviour which may lead to malnutrition and significant negative effects on social and occupational functioning [1]. ON tendencies have been measured in a variety of populations including medical students [2], nutritionists [3], dietitians [4], medical doctors [5], performance artists [6], athletes [7] and yoga practitioners [8]. Orthorexia nervosa does not appear to be age-related [9] and the evidence base with respect to gender disparities is at present equivocal [7, 10, 11].

The symptomatology of ON varies across the literature, but diagnostic criteria generally include severe dietary restrictions, excessive time spent on preparing and shopping for food, increased spending, preoccupation with purity and provenance of ingredients, eating alone, feelings of guilt if non-adherent, and perceived superiority over others when adherent [12]. With a significant increase of ON-specific issues being reported by eating disorder professionals [13], calls have been made for the formal inclusion of ON into diagnostic manuals [14].

Whilst it has been argued that ON is a unique and specific eating disorder [12, 15, 16], a number of studies have identified common affective and cognitive agents operating in ON, obsessive–compulsive disorder (OCD) and other eating disorders [17, 18]. More specifically, it has been suggested that anxious states similar to those reported by people with anorexia nervosa (AN) and OCD (strong beliefs about food and obsessive eating habits) may be characteristic of ON [19, 20] and lead to “health anxiety” [21]. Moreover, in contrast to body image and weight-related concerns, which are prevalent in AN and bulimia nervosa [22], over exercising and weight loss do not seem to be the main objectives of those experiencing ON, although unintentional weight loss might result from highly selective eating [23–25]. Interestingly, the presence or absence of ON does not seem to be associated with body mass index [26, 27]. There is also the tendency for those with ON to report the experience of positively valanced emotions associated with one’s body image, attachment style perfectionism, increased self-esteem and narcissism [28].

With the recent growth of the health-food sector, an ever-increasing presence of healthy eating promotional information in the mass and social media [29, 30] and evidence that more restrictive eating (such as that adopted in vegetarianism) may be associated with increased ON likelihood [31], understanding those psychological processes linked to the development and maintenance of ON-related behaviours is of increasing importance. One prediction suggests that, for example, that like other eating disorders, ON may be characterized by the inability to adapt thinking to newly encountered information/events. This cognitive inflexibility, however, was not shown to be characteristic of ON compared to controls [16, 32].

An alternative information processing bias that has been shown to play a fundamental role in a number of eating disorders concerns how attentional biases influence the processing of concern-related stimuli [33, 34]. This might be another candidate starting point for delineating characteristics of ongoing ON-related thought. Various cognitive models suggest that attentional bias helps to establish and maintain eating disorders and other forms of appetitive behaviours [34, 35] by inducing distractibility and attentional approach towards concern-related environmental cues [36–38], which after repeated exposure becomes unconscious [39]. This is comparable with hyper-vigilance and craving induced by automatic biased processing seen in substance dependence [39, 40], gambling [41] and various other compulsive and habitual health-related behaviours [40, 42].

Attentional bias research employs a variety of experimental paradigms (e.g. visual probe, eye tracking) with the modified Stroop task showing that people with eating disorders tend to respond slower than a control group to concern-related words in clinical and non-clinical populations [see 43, 44 for meta-analyses, 33, 45]. Importantly, recent evidence suggests that these effects are associated with ongoing related behaviours such as purging in bulimia nervosa [36]. Theoretically it has been argued that impaired attentional processing and appetitive responses to concern-related information may lead to the systematic strengthening of dopaminergic or “pleasure” neurobiological pathways and also craving-induced sensitization [39]. This may lead to increased saliency of interest-related cues and to a heightened “need” to consume, for example, particular food stuffs to increase positive and minimize negative affective states [46, 47]. To date, no work has examined the operation of attentional biases in relation to individual differences in ON-related psychological tendency. It would be plausible to expect, however, that people with an increased tendency towards ON may respond differently to food-related words relative to those with decreased ON tendencies. More specifically, it is predicted that those with increased ON tendencies will show interference to healthy-related words, a pattern of results not predicted for those with decreased ON tendencies. To explore the operation of preferential attention to food-related stimuli, the present study examined the relationship between ON tendencies and attentional biases for healthy and unhealthy related food words.

## Methods

### Design

The study used a correlational design with attentional bias scores (mean correct responses in milliseconds [ms]) derived from two modified food-Stroop tasks as independent variables and ORTO-15 scores as the dependent variable. Attentional bias (AB) scores were derived by subtracting mean correct reaction times (ms) for neutral words from mean correct reaction times for their matched healthy and unhealthy words (see Table 1 for means and standard deviations). Scores greater than zero represent increased interference from healthy/unhealthy words. Words for inclusion in the Stroop tasks were derived from a pilot study (see “Procedure” section).

**Table 1:** Millisecond mean correct reaction times (standard deviations in parentheses) and attentional bias scores for healthy, unhealthy and matched neutral food-related word types

Word type					
Healthy	Matched neutral	AB score	Unhealthy	Matched neutral	AB score
724.21 (132.32)	698.67 (148.50)	+ 25.54* (94.42)	707.02 (160.93)	723.42 (161.08)	− 16.40 (79.42)

*N* = 80

*p < 0.05 one-sample *t* test against zero

### Participants

Eighty participants (20 males and 60 females) aged between 18 and 58 years (mean age = 29.43; sd = 10.6) took part in the main study. Forty-five (56.3%) participants were recruited from undergraduate students at a London university in return for course credit, and the remainder (*n* = 35, 43.7%) were members of the public recruited from the local area.

### Materials

#### Deriving words for use in the healthy and unhealthy food Stroops

Two groups of five participants were recruited for a two-stage pilot phase and comprised individuals approached by the researcher at various locations such as health food stores and gyms in London. These individuals did not take part in the main study. Initially, in the first stage five individuals were asked to generate as many words related to the categories “healthy” and “unhealthy” food (categories were counterbalanced) as possible in 3 min. A second group of five individuals were then asked to rate these 48 words for their category representativeness and familiarity. The 48 words had been reported by at least three of the five participants in stage 1 (25 healthy and 23 unhealthy words). For each word participants were presented with the statements: “This word is related to healthy/unhealthy food and eating habits” and “I am familiar with this word” and asked to rate the extent to which they agreed or disagreed with each on a 5-point Likert scale (1 = strongly disagree to 5 = strongly agree). The next phase examined how the pilot participants rated the healthiness and desirability of each word using a 5-point Likert scale (1 = very healthy to 5 = very unhealthy and 1 = very undesirable to 5 = very desirable). All the words were presented to participants in a random order. Desirable unhealthy words and undesirable healthy words were discarded (e.g. raw). The final set of five words for each of the unhealthy and healthy word categories consisted of those rated the highest for familiarity, category representativeness, healthiness/unhealthiness and desirability for healthy and unhealthy words. The neutral category constituted words representing “things found in the office” [48] and were matched with the food-related words for their length and frequency. This resulted in the following categories: healthy food-related words—fresh, vegetables, whole, salad and natural; healthy food-matched neutral words—files, calculator, ruler, paper, and printer; unhealthy food-related words—processed, greasy, fried, salt, takeaway; unhealthy food-matched neutral words—envelope, pencil, chair, lamp, blinds.

#### ORTO-15

Participants also completed the ORTO-15 questionnaire comprising 15 items addressing cognitive, emotional and clinical factors arising from ON. For example: “Are your eating choices conditioned by your worry about your health status?”; “Is the taste of food more important than the quality when you evaluate food?”. Answers were given on a four-point Likert scale (1 = always to 4 = never). Low scores indicate increased ON tendency. ORTO-15 has been used to assess the prevalence of ON behaviours across a variety of clinical [7] and non-clinical populations [49].

#### Current hunger

Participants rated their current levels of hunger on a 5-point Likert scale (‘strongly disagree’ to ‘strongly agree)’ in response to two statements: “At the moment I feel hungry” and “At the moment I do not feel hungry” before and after the administration of the Stroop task. These variables were measured to ascertain any change pre- and post-Stroop administration.

### Procedure

Following consent, participants initially rated their current hunger levels and then completed the two modified Stroop tasks to measure attentional preference separately for unhealthy-related and healthy-related food words. Presentation order of Stroop type was counterbalanced across participants. For each experimental session, participants completed two blocks of practice trials (40 trials prior to each experimental block) in which non-word letter strings (e.g. XXXY, YYYY) were displayed in any one of four colours (blue, green, red, yellow). Participants then completed healthy and unhealthy food Stroops in a counterbalanced order. In each of the two Stroop tasks participants were presented with either healthy or unhealthy words, and their matched neutral healthy or matched neutral unhealthy words in each of the four colours. All stimuli were presented in blocks. Participants were asked to ignore the word and respond to the colour in which the word was presented by pressing one of four keys labelled with the colour name. Each word appeared in the centre of the computer screen until a key response was made at which point the next word appeared. Across a total of 120 trials within healthy/unhealthy and matched neutral blocks, words were presented randomly in each of the fours colours and repeated three times (60 trials for each of the healthy and unhealthy blocks and 60 trials for each of the matched healthy and matched unhealthy blocks). Total number of trials per participant was 240 trials. Words were presented using ePrime (Psychology Software Tools Inc., Pittsburgh, Pennsylvania) and conducted on a 13.3-in. Lenovo laptop with an LCD screen. Accuracy and reaction time (millisecond) for each word was recorded. Participants then completed the second current hunger questionnaire and the ORTO-15. Completion of these questionnaires after the Stroop tasks aimed to eliminate possible priming of food-related content on subsequent performance. All testing took place in a single occupancy quiet room or experimental cubicle.

## Results

Initial analysis of the overall sample (*N* = 80) showed that ORTO-15 scores ranged between 27 and 45 (mean = 36.93; sd = 4.45). An independent samples *t* test revealed no significant differences between males and females (*t* (78) = 1.030, *p* = 0.88), nor any correlation between age and the ORTO-15 scale scores (*r* = 0.02, *p* = 0.151). In addition, across all participants the mean AB score for healthy food-related words (mean = 25.54, sd = 94.42) was shown to differ significantly from zero (the point of no difference in response speeds to these stimuli), *t* (79) = 2.42, *p* < 0.05. This was not shown for mean AB score for unhealthy food-related words, *t* (79) = 1.85, *p* > 0.05. The size of the bias for healthy words was also significantly greater than for unhealthy words, *t* (79) = 3.23, *p* < 01.

To examine the predictive relationship between attentional bias scores above and beyond current hunger status and ORTO-15 scores for the sample as a whole and for those scoring below the ORTO-15 cut-off of 40 (indicative of increased ON tendencies), hierarchical multiple regressions were used. In each regression ORTO-15 scores were the criterion factor with change in hunger level entered at the first step and attentional bias scores at the second step. Prior to analyses all assumptions for the use of multiple regression were assessed.

### Predicting ORTO-15 scores

Pearson’s *r* correlational coefficients were calculated between the criterion variable and possible predictor variables for justification of inclusion in the analysis. Only those variables significantly correlated with ORTO-15 were included in the main analysis (see Table 2). Prior to the hierarchical multiple regression, relevant assumptions for this test were examined. First of all, a sample size of 80 was adequate given two independent variables entered into the analysis [50]. Table 2 shows that the two variables shown to correlate with ORTO-15, change in hunger levels pre- and post-testing and AB scores for healthy food-related words, were not correlated with each other and collinearity statistics were within acceptable limits suggesting low multicollinearity—tolerances > 0.10; average VIF < 10. Mahalanobis distance scores showed there to be no multivariate outliers and residual and scatterplots suggested that normality, linearity and homoscedasticity assumptions were met.

**Table 2:** Pearson’s *r* correlational coefficients for current hunger change score, AB for healthy and unhealthy-related words and ORTO-15 scores

	Hunger change	AB healthy	AB unhealthy
ORTO-15 score	− 0.33**	− 0.26*	0.03
Hunger change		0.04	0.15
AB healthy			0.12

*N *= 80

*p < 0.05, **p < 0.01

We then used a hierarchical multiple regression to examine the effects of attentional bias scores on ORTO-15 scores controlling for change in hunger levels pre- and post-testing. Specifically, change in hunger level was entered at the first step in predicting ORTO-15 and AB scores for healthy food-related words added to the equation in the second step. At step one, change in hunger level was shown to significantly predict ORTO-15 scores, *F* (1, 78) = 9.21, *p* < 0.01, *R*^2^ = 0.11, adj *R*^2^ = 0.09. At step 2, adding AB scores for healthy food-related words to change in hunger level also significantly predicted ORTO-15 scores, *F* (2, 77) = 7.61, *p* < 0.001, *R*^2^ = 0.17, adj *R*^2^ = 0.14. The addition of AB scores for healthy food-related words significantly increased the proportion of variance explained in ORTO-15 scores, *∆F* (1, 77) =5.48, *p *< 0.05, *∆R*^*2*^ = 0.06. When both predictor variables were included in the equation both change in hunger level (β = − 0.32, 95% CIs [− 3.85, − 0.79]) and AB scores for healthy food-related words (β = − 0.24, 95% CIs [− 0.02, − 0.002]) were shown to both predict ORTO-15 scores (*p*s < 0.05) and uniquely accounting for 10% and 6%, respectively, of the variation in ORTO-15 scores (see Table 3).

**Table 3:** Summary of hierarchical regression analysis for variables predicting ORTO-15 scores

Variable	β	*t*	sr^2^	*R*	*R*^*2*^	*∆R*^*2*^
Step 1				0.33	0.11	0.11
Change in hunger	− 0.33	3.04**	0.11			
Step 2				0.41	0.17	0.06
Change in hunger	− 0.32	3.03**	0.10			
AB healthy food words	− 0.24	2.34*	0.06			

*N* = 80

*sr*^*2*^ semi-partial correlation^2^

*p < 0.05, **p < 0.01

### Predicting ORTO-15 scores less than 40

Our final analyses examined the relationship between ORTO-15 scores as a criterion factor and relevant predictor variables among those scoring either above (*n* = 56) or below 40 (*n* = 24) on the OTRO-15 as a threshold for ON risk [see 51]. For those scoring below 40 (higher in ON risk), initial Pearson’s *r* correlations showed ORTO-15 to be significantly associated with change in hunger status (*r* = − 0.34, *p* < 0.01) and AB scores for healthy words (*r* = − 0.31, *p* < 0.05) but not AB for unhealthy words (*r* = − 0.01, *p* = 0.95). The sample size of 50 was adequate given two independent variables entered into the analysis [50]. An assessment of assumptions for linear regression analysis showed that the relationship between change in hunger levels and AB scores for healthy food-related words were not correlated (*r* = − 0.01, *p* = 0.98), collinearity statistics were within acceptable limits suggesting low multicollinearity (tolerances > 0.10; average VIF < 10), Mahalanobis distance scores showed there to be no multivariate outliers, and residual and scatterplots suggested that normality, linearity and homoscedasticity assumptions were met. The same regression analysis as that reported previously was then performed for the sub-sample.

At step one, change in hunger level was shown to significantly predict ORTO-15 scores, *F* (1, 54) = 6.89, *p* < 0.05, *R*^2^ = 0.11, adj *R*^2^ = 0.10. At step 2, adding AB scores for healthy food-related words to change in hunger level also significantly predicted ORTO-15 scores, *F* (2, 53) = 7.10, *p* < 0.01, *R*^2^ = 0.21, adj *R*^2^ = 0.18. The addition of AB scores for healthy food-related words significantly increased the proportion of variance explained in ORTO-15 scores, *∆F* (1, 53) =6.59, *p *< 0.01, *∆R*^*2*^ = 0.10. When both predictor variables were included in the equation change in hunger level (β = − 0.34, 95% CIs [− 3.13, − 0.499]) and AB scores for healthy food-related words (β = − 0.31, 95% CIs [− 0.02, − 0.002]) were shown to both predict ORTO-15 scores (*p*s < 0.01) and uniquely accounted for 12% (sr = − 0.34) and 10% (sr = − 0.31), respectively, of the variation in ORTO-15 scores.

Regression analysis for those scoring above 40 (lower in ON risk) was not performed because initial Pearson’s *r* correlation showed ORTO-15 to correlate significantly with AN for unhealthy words (*r* = − 0.53, *p* < 0.10), but not with change in hunger (*r* = 0.18, *p* = 0.39) nor AB for healthy words (*r* = 0.01, *p* = 0.98).

## Discussion

This study employed a modified food-Stroop task to assess the relationship between attentional preference for healthy/unhealthy food-related words over matched neutral words and ON tendency scores in a non-clinical sample of individuals. ON tendencies was based on responses to the ORTO-15 [51], a measure previously used to assess the prevalence of ON-related behaviours across a variety of clinical and non-clinical populations [7, 49]. Results showed that, in general, individuals showed a significant attentional preference for healthy food-related words and not unhealthy food-related words, and that the magnitude of this bias was significantly greater for healthy relative to unhealthy words. Participants showed significantly greater interference from healthy food-related words relative to unhealthy food-related words. This suggests that the general pattern of responding, irrespective of ON tendencies, is for people to show a pattern of responding consistent with an attentional bias for healthy food-related words and that this bias is larger when compared with responses to unhealthy food-related words.

Our next question was to ask whether this pattern of responding was associated with individual differences in ON tendencies. The initial prediction was that relative to participants with decreased ON tendencies, people with higher ON tendencies would show an increased attentional bias for food-related healthy words and not for unhealthy food-related stimuli. This was supported in the current study. Only AB for healthy food-related words were found to be significantly associated with ORTO-15 scores. The negative correlation shows that increased ON tendencies are associated with increased attentional preference for healthy food-related words. Our findings also showed that attentional preference for healthy food-related words is a significant independent predictor of ON tendencies over and above other variables. In our study, we measured levels of hunger pre- and post-modified Stroop to control for the idea that performing the Stroop itself could act as a prime for beliefs about one’s hunger to the extent that this then accounts for any variability in reported ON tendencies. The hierarchical regression analysis did indeed show that change in hunger status was significantly predictive of ON tendencies with increased ON tendencies associated with increased hunger post-Stroop. However, it also highlighted that attentional bias scores for healthy food-related words added significantly more variance explained in ORTO-15 than that explained by change in hunger status alone. Moreover, the proportion of unique variance explained by AB for healthy food-related words was significant (β = − 0.24, sr^2^ = 0.06). Notwithstanding debate around the categorization of ON via various psychometric measures (see [62]), that we derived an identical set of findings among those who would be deemed as ON according to recommended ORTO-15 scores [51], but not for those who scored above this threshold is not without significance. Indeed, the amount of *unique* variance accounted for by AB for healthy words was shown to increase (β = − 0.31, sr^2^ = 0.10). This shows that the relationship between increasing AB for healthy food-related stimuli and ON tendencies is magnified further among those who may already be so characterized. The question as to whether this relationship is linear or more curvilinear in nature requires future investigation. One possibility is that this relationship is an incremental increase in both AB and ON severity. An alternative possibility is that increasing AB is more crucial in the earlier stages of ON severity compared to a more chronic ON phase.

Overall, this evidence suggests that cognitive markers of attentional preference for healthy food-related words are incrementally discriminative with increasing ON-related tendencies (i.e. ORTO-15 scores). These findings are novel in the ON literature and adhere to the pattern of findings found utilizing a modified Stroop task to assess responses to concern-related stimuli in overweight and normal-weight individuals. In essence, these groups show elevated attentional bias for food-related words [52, 53], which may predict subsequent weight gain [54].

Moreover, our evidence is consistent with the general idea that people tend to show increased attentional preference for stimuli that are of more “concern” for them and that these information processing biases occur during ongoing experience a position reflected in empirical evidence and theoretical approaches for understanding numerous behaviours [36, 38, 55, 56]. These information processing accounts argue that with repeated behavioural experience concern-related stimuli are detected automatically (without conscious awareness) and may trigger desire-related thoughts and ongoing behavioural patterns [39, 40]. In the case of individuals with higher tendencies towards ON, it is likely that with repeated experience of relevant behaviour, healthy food-related stimuli are more likely to catch the attentional system (i.e. are more salient for processing) relative to non-concern-related stimuli and that such attentional processing operates outside of one’s immediate awareness. We are not, however, stating any causal relationship. Our data suggest a relationship only and future prospective work or experimental manipulation of such biases is required to determine causality. For instance, studies which have either (1) manipulated such biases through intervention and measured subsequent behaviour [57, 58] or (2) prospectively predicted relapse behaviour from known bias levels [59, 60] for numerous addictive behaviours show equivocal evidence. On this basis, it has been argued that attentional bias may not be a stable intrinsic characteristic which influences behavioural enactment processes but should be considered as operating in-the-moment immediately prior to behaviour [61].

The current study also showed that ON tendency was not associated with response patterns to unhealthy words relative to neutral controls to the extent that there was no statistically significant interference from these stimuli. A priori it may have been expected that such an interference would have been shown with increasing ON tendency because these stimuli may be more “threatening” in nature and to be avoided [38, 61]. That this was not the case argues against this perspective. It seems that among those with increasing ON tendencies, the attentional system is more finely tuned to the extent that it is specific to healthy food-related stimuli.

An alternative explanation for the operation of the bias towards healthy and not unhealthy food-related words as a function of increasing ON tendencies proposes that whilst healthy stimuli are of concern this concern may best be reflected as a form of desire. Whilst desire was not explicitly measured, we did measure beliefs about changes in levels of hunger pre- and post-Stroop task. If we assume that degree of in-the-moment hunger acts as a proxy for desire, we would expect a significant association between healthy food-related interference and change in hunger experienced. In other words, attentional biases should trigger feelings or thoughts associated with hunger. This was not found to be the case. Both in-the-moment change in hunger and AB for healthy food-related words independently predicted ORTO-15, with AB for healthy words adding significantly explained variance in ORTO-15 beyond that explained by change in hunger alone. To test this idea further future work is required to assess more precisely the desire/urge responding for healthy food-related stimuli among those with increased ON tendencies and how information processing biases (approach–avoidance bias, attentional bias, etc.) may influence this relationship.

These study findings may be tempered by a number of apparent limitations. First of all, whilst the ORTO-15 is a widely used measure of ON tendencies, recent debate has questioned its psychometric properties [62]. Future work should examine the effect of information processing biases using alternative measures of ON. Three possible candidates are the Eating Habit Questionnaire (EHQ) [63], Düsseldorfer Orthorexia Scale (DOS) [64] and the Teruel Orthorexia Scale (TOS) [65]. Validation of each approach as a function of attentional preference for relevant stimuli would be a useful addition to the literature. However, given that recent work argues for the dissociation in food choice motivations between so-called healthy ON (an interest in healthy eating) and ON (a pathology characterized by the pre-occupation with healthy eating) [66], and that this distinction is articulated fully in the development of the TOS, future work should examine the nature of any observed bias in information processing as further evidence for any possible cognitively based indices. A second potential limitation refers to the fact that the stimuli used in the Stroop task may not fully match individual differences in the importance ascribed to these stimuli. It is possible that those with more pronounced ON are “influenced” by a different set of stimuli compared to those with a less pathological profile. Finally, whilst our findings suggest an attentional preference for healthy food-related words, other measures of attentional bias and allocation of attentional resources should be adopted in future work. For example, use of eye-tracking technology would enable the study of how individuals with and without an ON profile initially orientate attention towards relevant stimuli and whether these individuals are able to disengaged from such stimuli once attended to. Such work would provide useful insights for articulating a cognitive understanding of ON tendencies.

Overall, it appears that attentional preference for healthy food-related stimuli is characteristic of individuals with increasing ON-related tendencies. That such attentional preference adds significant additional explanatory power above any effects observed for change in hunger status suggests an independent relationship. Future work is required to replicate and validate this finding utilizing alternative measures of attentional allocation (e.g. dot probe tasks, eye-tracking technologies) and to examine, for example, initial orientation and gaze maintenance/delayed disengagement processes as further potential discriminatory factors.
